# Oncogenic FGFR Fusions Produce Centrosome and Cilia Defects by Ectopic Signaling

**DOI:** 10.3390/cells10061445

**Published:** 2021-06-09

**Authors:** Alexandru Nita, Sara P. Abraham, Pavel Krejci, Michaela Bosakova

**Affiliations:** 1 Department of Biology, Faculty of Medicine, Masaryk University, 62500 Brno, Czech Republic; nitaa@mail.muni.cz (A.N.); sara.abraham@med.muni.cz (S.P.A.); krejcip@med.muni.cz (P.K.); 2 Institute of Animal Physiology and Genetics of the CAS, 60200 Brno, Czech Republic; 3 International Clinical Research Center, St. Anne’s University Hospital, 65691 Brno, Czech Republic

**Keywords:** FGFR, fibroblast growth factor receptor, FGFR fusion, cancer, oncogenic driver, neoplastic transformation, primary cilia, cilia, centrosome, centrosome cycle

## Abstract

A single primary cilium projects from most vertebrate cells to guide cell fate decisions. A growing list of signaling molecules is found to function through cilia and control ciliogenesis, including the fibroblast growth factor receptors (FGFR). Aberrant FGFR activity produces abnormal cilia with deregulated signaling, which contributes to pathogenesis of the FGFR-mediated genetic disorders. FGFR lesions are also found in cancer, raising a possibility of cilia involvement in the neoplastic transformation and tumor progression. Here, we focus on FGFR gene fusions, and discuss the possible mechanisms by which they function as oncogenic drivers. We show that a substantial portion of the FGFR fusion partners are proteins associated with the centrosome cycle, including organization of the mitotic spindle and ciliogenesis. The functions of centrosome proteins are often lost with the gene fusion, leading to haploinsufficiency that induces cilia loss and deregulated cell division. We speculate that this complements the ectopic FGFR activity and drives the FGFR fusion cancers.

## 1. Primary Cilium and Its Role in Cancer Development

A majority of the vertebrate cells are capable of forming a primary cilium, a microtubule-based organelle that projects from the centrosome to integrate signaling pathways and mediate cell-to-cell communication. Mutations in genes that control cilia structure or function produce a growing list of diseases called ciliopathies. To this day, at least 35 ciliopathies exist, and more than 400 candidate proteins have been identified [1]. Virtually all annotated ciliopathies are genetic developmental disorders; however, function of cilia in the tissue homeostasis is also beginning to emerge [2].

During cell division, the centrosomes need to function in the mitotic apparatus. Therefore, the cilium is typically disassembled during mitosis, even though cilia rudiments may be preserved [3,4]. The presence of a primary cilium is, therefore, tightly coupled with the cell cycle. In the majority of the cilia-competent cells, the primary cilium is formed during the G0/G1 phase of the cell cycle and resorbs before the S phase [5,6]. Several mitotic kinases, Aurora A [7,8], polo-like kinase 1 (PLK1) [9] and NIMA-related kinase 2 (NEK2) [10], were shown to block assembly and induce disassembly of the primary cilium, and upregulated activity of these kinases is frequently found in cancer [11,12,13,14,15,16,17,18,19]. Inhibition of the cilia disassembly signaling using small chemical inhibitors restored ciliogenesis and suppressed tumor growth in cholangiocarcinoma [20] or chondrosarcoma [21].

It is mainly the loss of primary cilia, as well as of their regulatory function in cellular signaling and cell division, that has been associated with neoplastic transformation and tumor progression [22,23,24,25]. In glioblastoma, disruption of ciliogenesis was observed at all stages, starting at early tumor lesions [26]. In a mouse model of Kirsten rat sarcoma virus protein (Kras)-driven pancreatic cancer, neoplastic lesions were coupled with cilia loss [27], and a similar observation was in precursor lesions of pancreatic cancer patients [27,28]. In breast cancer, inhibited ciliogenesis was reported within the tumor tissue [29,30,31]. Importantly, in a mouse model of breast cancer, genetic ablation of primary cilia led to earlier tumor formation, faster tumor growth rate, and increased metastasis [32]. Reduced ciliation has also been associated with the onset of prostate cancer [33], rhabdomyosarcoma [34] or chondrosarcoma [35], altogether supporting the role of primary cilia as tumor suppressors.

The Hedgehog (Hh) pathway plays fundamental roles in tissue morphogenesis and homeostasis [36,37,38,39,40,41,42,43,44], and is frequently activated in cancer [45,46]. In vertebrates, the canonical Hh signaling depends on primary cilium. Briefly, activation of the pathway allows for ciliary accumulation of Smoothened, which is accompanied by posttranslational activation of the effector transcription factors from the glioma family, Gli2 and Gli3, within the cilia, and induction of the target genes [47,48,49,50,51]. In Hh-addicted cancers such as medulloblastoma and basal cell carcinoma, the presence of a primary cilium can both promote and suppress tumorigenesis, depending on the oncogene identity. The following studies introduced this paradigm. In a mouse model of medulloblastoma, conditional expression of a constitutively active Smoothened variant SmoM2 leads to tumor formation. Genetic ablation of cilia in the SmoM2-expressing cells completely blocked medulloblastoma formation [52]. Mice harboring only one copy of the cilia-resident Hh pathway inhibitor Patched also develop medulloblastoma, which is abrogated by conditional deletion of cilia [53]. Another medulloblastoma mouse model depends on ectopic expression of the Hh effector Gli2. Tumor development in these mice, however, occurs only after conditional removal of primary cilia, as the cilia presence effectively reduced the Gli2 activity [52]. Notably, similar conclusions were obtained in the Hh-driven basal cell carcinoma. Abundant ciliogenesis was found in patient biopsies and primary lesions in a mouse model constitutively expressing SmoM2 in keratinocytes [54]. Removal of primary cilia abolished tumor development in SmoM2 animals, but accelerated cancerogenesis in mice with conditional expression of active Gli2.

Persistent or increased ciliation has also been associated with other types of cancer. In the choroid plexus, the ectopic presence of Hh-responsive cells harboring a primary cilium produced neoplasm in the mouse [55]. During epithelial–mesenchymal transition of the mammary cancer stem cells, the ciliation increases together with tumorigenic properties of the transplanted cells. Epigenetic or chemical ablation of cilia inhibited Hh signaling and tumorigenic ability of these cells [56]. Taken together, the initiation and progression of the Hh-driven cancers takes advantage of the primary cilium if that is needed to achieve the oncogene activity.

## 2. FGFR Regulates Cilia Motility and Signaling during Morphogenesis

The fibroblast growth factor receptors (FGFR) have a well-recognized function in the regulation of cilia. Four members of the FGFR family exist, denoted as FGFR1-4 [57,58,59,60], and respond to at least 18 secreted FGF ligands [61,62,63] by dimerization, transactivation and engagement of multiple intracellular signaling pathways [63,64,65,66]. The FGF–FGFR interaction is facilitated by the low affinity co-receptors, i.e., heparan sulfate proteoglycans for most FGF ligands that signal in a paracrine fashion, and Klotho proteins for endocrine FGF19, FGF21 and FGF23 [67,68,69,70,71,72,73,74,75,76,77,78,79]. FGFRs regulate a variety of physiological processes, including morphogenesis [80,81,82,83,84,85,86,87], metabolism [88,89,90,91,92,93] and regeneration [94,95,96,97,98]. Consequently, disrupted FGFR signaling manifests in a plethora of pathological conditions such as developmental ciliopathies [99,100,101] and cancer [70,102,103,104,105,106].

Mounting experimental evidence points towards a functional relationship between FGFR signaling and cilia. In the *Xenopus* organ of laterality, the gastrocoel roof plate, shorter cilia were obtained after expression of dominant-negative Fgfr1. In zebrafish, morpholino knockdown of *fgfr1*, expression of dominant-negative Fgfr1, treatment with FGFR kinase inhibitor or loss of *fgf4*, *fgf8* or *fgf24* all reduced cilia length in Kupffer’s vesicle and perturbed the cilia-mediated directional fluid flow that is required for left-right patterning of the zebrafish embryo [107,108,109]. In a follow-up study, the zebrafish *fgfr2c* morphants had shorter cilia in the Kupffer´s vesicle, and showed multiple developmental defects coupled with abnormal left-right polarization, including randomized positioning of the liver and pancreas, disrupted heart looping, and defective brain morphogenesis [110]. A similar phenotype was observed in *Xenopus* with depleted *fgfr4* [111]. Morpholino knockdown of the zebrafish FGF target genes *ier2* and *fibp1*, or of the proteoglycan sulfotransferase 3-OST-5 also shortened cilia in the Kupffer´s vesicle and induced laterality defects [112,113], and this was associated with lower expression of genes important for ciliogenesis [107,113,114,115,116]. Taken together, the cilia length and motility within the organ of laterality is regulated by FGFR signaling, which is critical for establishment of the left-right body asymmetry.

The FGFR signaling also regulates cilia during the later stages of development. Injection of a FGFR kinase inhibitor into neonatal mice produced cilia shortening in the biliary duct, proximal kidney tubules and lungs [99]. The zebrafish *fgfr1* morphants had shorter tethering cilia in the otic vesicle and motile cilia in the pronephric ducts [107]. In the inner ear mechanosensory hair cells, FGFR1 localizes to kinocilia and regulates its length and stability [117]. In cultured mammalian cells, a ligand-mediated FGFR activation elongated primary cilia, via accelerated ciliary transport [99,118]. This was coupled with reduced ciliary Smoothened trafficking and inhibited Hh signaling. The molecular mechanism of the FGF-mediated cilia elongation involves ERK MAP (extracellular signal-regulated kinase mitogen-activated protein) kinase and mechanistic target of rapamycin complex 1/2 (mTORC1/2) pathways [99], and the phosphorylation-mediated inactivation of the conserved cilia regulator kinase CILK1 (ciliogenesis associated kinase 1) [101,119,120,121,122,123,124,125,126]. These data further connect the FGFR signaling with the cilia functions.

## 3. Aberrant FGFR Signaling Affects Primary Cilia

Pathological FGFR activity has been associated with shortening of primary cilia [99,100]. Gain-of-function missense mutations in FGFR3 produce human skeletal dysplasias, including achondroplasia and thanatophoric dysplasia [127,128,129,130,131], and frequently occur in cancer [132,133,134,135,136]. Several studies pointed towards a cross-talk of FGFR3 signaling with the cilia-associated Hh pathway that was found inhibited in mouse models of achondroplasia [137,138,139], due to the defective ciliogenesis [99,100]. Shorter cilia were also found in the cartilage of humans with thanatophoric dysplasia, and in cells overexpressing a pathological FGFR3 variant [99,100]. In cultured cells, pathological FGFR3 activity inhibited the Hh pathway, reduced ciliary Smoothened trafficking, and shortened cilia, possibly via reduced ciliary transport which limited the tubulin flux necessary for cilia maintenance [99,100,140]. FGFR kinase inhibitors normalized the cilia length in vitro [99,100], and Hh signaling in the cartilage in vivo [107,141]. Taken together the pathological FGFR activity interfered with ciliogenesis and cilia function. This was in part due to increased Aurora A and PLK1 activity [12,142,143], that are also found upregulated in the FGFR1-driven cancers [144,145,146]. Therefore, it is likely that the FGFR cancers are driven, at least partly, by cilia disassembly that alleviates the mitotic brakes and increases availability of centrosomes for the mitotic spindles [147,148].

## 4. FGFR Gene Fusions in Cancer

Deregulated FGFR signaling, mostly caused by increased FGFR activity, has been implicated mainly in tumor progression, through poorly understood mechanisms involving accelerated proliferation, resistance to apoptosis and enhanced angiogenesis [93,149,150,151,152]. Among the 4853 tumor samples analyzed by next generation sequencing, a *FGFR* aberration was found in 7.1% of all cases [153]. The most frequent lesion was gene amplification, accounting for 66% of *FGFR* aberrations [153], and typically resulting in FGFR overexpression and increased activity [154,155,156,157,158]. *FGFR* mutations were less frequent, covering 26% of the identified aberrations [153]. More than 200 distinct *FGFR* point mutations have been identified in cancer, targeting the extracellular, transmembrane and kinase domains of all four FGFRs [133,159,160,161]. The majority of the mutations lead to ligand-independent FGFR dimerization and increased pathway activity [162,163,164,165]. Interestingly, somatic mutations found in cancer frequently overlap with those causing developmental disorders (extensively reviewed in [133]); however, increased incidence of tumors has not been reported in these disorders. This can be exemplified by activating FGFR3-K650E/M mutation, causing thanatophoric dysplasia type II and SADDAN (severe achondroplasia with developmental delay and acanthosis nigricans), respectively [128,129,166,167]. Although this mutation has been detected in aggressive cancers, it failed to induce neoplastic transformation in mice. Additional mutation, involving deletion of the tumor suppressor PTEN (phosphatase and tensin homolog) or activating KRAS mutation were required to induce the FGFR3 cancerogenesis [168,169]. These data suggest that *FGFR* missense mutations are not likely to initiate the neoplastic transformation, but rather occur later to promote tumor progression and metastasis.

**Figure 1:**
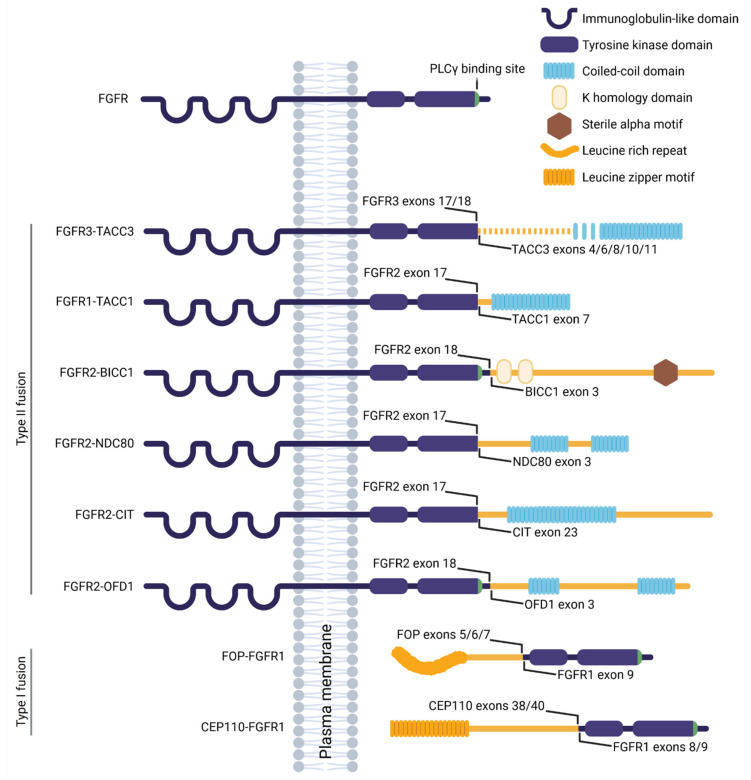
Schematic representation of the FGFR fusion proteins. The wild-type FGFR comprises the extracellular immunoglobulin-like domain, responsible for ligand binding, the transmembrane region, and the intracellular part that is responsible for binding and activation of the signal transducers including PLCγ (binding site indicated in green). The type II fusions lose a variable part of the C-terminal region of FGFR, frequently involving the PLCγ binding site, and attach a truncated C-terminal part of the fusion partner. In type I fusions, the FGFR extracellular and transmembrane parts are excluded, and the truncated fusion partner joins in just before the FGFR kinase domain. In both types of FGFR fusion, the partner possesses domains that facilitate dimerization—the coiled-coil domain, the sterile alpha motif, the leucine rich repeat or the leucine zipper. The positions of the fusion breakpoints are indicated.

A gene fusion originates from the chromosomal rearrangement involving two genes, and results in a fusion protein capable of neoplastic transformation and oncogene addiction [170,171]. FGFR fusions are relatively rare, accounting for 8% of all *FGFR* aberrations found in cancer [153,172]. Additional missense mutations are sporadic [172], suggesting that the FGFR fusion protein holds sufficient oncogenic properties. In type I fusions, typically driving the hematological malignancies [173], the FGFR extracellular and transmembrane domains are excluded, and the fusion occurs at the N-terminus of the FGFR kinase domain (Figure 1). In type II fusions that are mostly found in solid tumors [173], the breakpoint usually occurs between exons 17 and 19, affecting only a varying part of the C-terminal region of FGFR [133]. In both types of fusion, the partner typically contains domains that facilitate dimerization such as the coiled-coil domain, the sterile alpha motif, the leucine rich repeat or the leucine zipper, leading to ligand-independent FGFR dimerization and signaling activity. The FGFR fusion protein may also be sequestered to an alternate subcellular location, trough features gained via the fusion partner, which can result in misplaced and deregulated activity. Finally, a substantial part of the fusion partner is typically lost during chromosomal rearrangement, producing haploinsufficiency or gaining novel function that may contribute to neoplastic transformation.

A substantial portion of the FGFR fusion partners are proteins associated with the centrosome functions, including spindle organization and ciliogenesis (8 of 14 recurrent FGFR fusions with at least partially characterized signaling properties; based on PubMed search in April 2021). This led us to speculation that disruption of the centrosome cycle may drive pathogenesis of the FGFR fusion cancers. In the following sections, we review the current knowledge of such oncogenic FGFR fusions, and discuss the possible involvement of both fusion partners in cancerogenesis. For a complete reference, the recurrent and characterized, yet not included fusions comprise FGFR2-CCDC6 [149,174], FGFR2-AHCYL1 [175,176], FGFR2-PPHLN1 [177,178], FGFR3-BAIAP2L1 [136,179,180], ZMYM2-FGFR1 [181,182,183], and BCR-FGFR1 [182,183,184].

### 4.1. FGFR3-TACC3

Gene fusion involving *FGFR3* and the transforming acidic coiled-coil containing protein 3 (TACC3) is one of the recurrent gene fusions, found in glioblastoma (29 of 103), non-small-cell lung carcinoma (28 of 103), head and neck squamous cell carcinoma (11 of 103), bladder cancer (10 of 103), and other types of cancer (Table 1) [133,149,153,179,185,186,187,188,189,190,191,192,193,194,195,196,197,198,199,200]. FGFR3-TACC3 transformed NIH3T3 and Rat1A fibroblasts [179,187,201,202], and the xenografted astrocytes or glioblastoma cells stably expressing FGFR3-TACC3 gave rise to gliomas [187,203]. Mice with hippocampal cells transduced with FGFR3-TACC3 developed invasive, rapidly growing high-grade gliomas [187], proposing FGFR3-TACC3 as an oncogenic driver.

The chromosomal rearrangement produces loss of the *FGFR3* 3′UTR containing miR-99a that normally regulates the FGFR3 levels; this leads to overexpression of FGFR3-TACC3 [203] and abundant transactivation of the FGFR3 residues [201]. Similar to the majority of the type II FGFR fusions, the FGFR3-TACC3 protein lacks the C-terminus of FGFR3 that is necessary for phospholipase C γ (PLCγ) binding (Figure 1), leading to silencing of this signaling branch [179,249]. Conversely, the ERK MAP kinase and STAT (signal transducer and activator of transcription proteins) signaling is increased in FGFR3-TACC3 expressing cells [201,203], and silencing of these pathways was partially successful in targeting the oncogene-driven growth of cell lines and xenografts [36,149,186,187,202,203,250,251,252].

TACC3 is an important component of the mitotic spindles, ensuring proper attachment of chromosomes to the microtubules [253,254]. During mitosis, FGFR3-TACC3 mislocalizes to the spindle poles while sequestering also the endogenous TACC3 from the mitotic spindle, through interaction of their coiled-coil domains [188,255,256]. This delays mitotic progression, and induces chromosome segregation defects and aneuploidy that increases by greater than 2.5 fold [187]. Interestingly, targeting TACC3 proved a viable strategy in TACC3-overexpressing cancers, likely by inducing abundant multipolar spindles, which led to mitotic arrest and apoptosis [257,258,259]. Elevated cellular levels of TACC3 were shown to induce loss of primary cilia through Aurora A induction and disruption of the transmembrane protein 67 (TMEM67)-filamin A complex [260,261], and promoted oncogenic transformation and shortened survival of the patients with prostate cancer [262]. Knockdown of TACC3 rescued ciliogenesis, reduced transformation and inhibited xenograft growth [262]. Taken together, FGFR3-TACC3 could lead to neoplastic transformation partly through induction of cilia disassembly and deregulated cell division, which are both druggable targets.

### 4.2. FGFR1-TACC1

The FGFR1 fusion with transforming acidic coiled-coil containing protein 1 (TACC1) was found in various types of tumors arising within the central nervous system (14 of 15; Table 1) [186,187,204,205,206,207,208,209,210,211]. FGFR1-TACC1 transformed C3H10T1/2 and Rat1A fibroblasts [187,263], and the xenografted astrocytes stably expressing FGFR1-TACC1 gave rise to gliomas [187].

The biological and oncogenic functions of FGFR1-TACC1 appear similar to those assigned to FGFR3-TACC3 [187]. TACC1 has a coiled-coil domain at the C-terminus, that is preserved in the fusion protein (Figure 1), and that mediates localization to the mitotic spindle [264,265,266]. FGFR1-TACC1 expression increased the rate of errors in chromosomal segregation about five times [187], likely through mislocalization and sequestration of endogenous TACC1, and similar spindle defects were observed in HeLa cells with depleted TACC1 [266]. TACC1 interacts with Aurora A, which appears critical for spindle formation, and the expression levels of the two proteins seem to correlate in cancers [266]. This suggests that TACC1 overexpression caused by FGFR1-TACC1 fusion could participate in neoplastic transformation through deciliation caused by increased Aurora A activity and deregulated cell division, similar to FGFR3-TACC3 cancers.

### 4.3. FGFR2-BICC1

About 45% of the intrahepatic cholangiocarcinoma cases are coupled with FGFR2 fusion, half of which are with bicaudal C1 (BICC1) [149,175,177,212,213,214,215]; identification of FGFR2-BICC1 in other types of cancer is rare [175] (Table 1). FGFR2-BICC1 transformed NIH3T3 cells that formed tumors in mice [175], and the xenografted FGFR2-BICC1 expressing liver organoids gave rise to tumors [267].

As a consequence of the chromosomal rearrangement, the *FGFR2* 3´UTR is truncated which results in upregulation of the FGFR2-BICC1 fusion protein [214]. FGFR2-BICC1 dimerizes likely via the sterile alpha motifs of BICC1 [268], leading to ligand-independent dimerization [149] and activation of the ERK MAP kinase, but not STAT3 or AKT signaling [175,212,267]. FGFR inhibitors were partially successful in targeting the oncogene-driven growth of cell lines, xenografts and patients’ tumors [175,215,269,270]; acquired resistance through gatekeeper FGFR2-V564F mutation was also reported [270]. The FGFR2^V546F^-BICC1 cells showed oncogene addiction that was fully inhibited by a synergistic effect of the FGFR and ERK MAP kinase pathway inhibitors [267].

BICC1 is a conserved RNA-binding protein that represses translation of selected mRNAs to control development [271,272,273,274,275]; the domains responsible for RNA binding are, however, partly lost during the chromosomal rearrangement, suggesting that this function is lost with the FGFR2-BICC1 fusion. Deletion of BICC1 leads to classical ciliopathy features, including randomization of the left-right asymmetry, and cystic development in the kidney, liver and pancreas [276,277,278,279,280,281,282,283]. Loss of BICC1 disrupted alignment of motile cilia and establishment of the cilia-driven fluid flow in the mouse embryonic node and *Xenopus* gastrocoel [279], producing laterality defects. This may be due to disrupted protein synthesis machinery at the centrosome that appears important for the adjacent cilia [284,285]. In humans, mutations in BICC1 were identified in patients with kidney dysplasia, likely caused by ectopic Wingless-related integration site (WNT)/β-catenin signaling [286]. Decreased levels of BICC1, or loss of some of the three RNA-binding domains which are also relevant for the FGFR2-BICC1 fusion, also upregulated WNT/β-catenin signaling [275,279,287,288,289]. Taken together, the FGFR2-BICC1 fusion is likely to produce a BICC1 haploinsufficiency that leads to disrupted ciliogenesis and cilia-associated signaling, which may contribute to cancerogenesis.

### 4.4. FGFR2-NDC80

A cholangiocarcinoma patient was described with a fusion comprising FGFR2 and NDC80 (or HEC1, highly expressed in cancer 1) [216]. FGFR2-NDC80 was overexpressed in the tumor cells, and activated the ERK MAP kinase, PLCγ, and STAT3 signaling [216]. Considering the PLCγ binding site is lost with the fusion (Figure 1), it is possible that FGFR2-NDC80 activates this pathway through heterodimerization with the endogenous FGFR. The fusion protein retains the kinetochore microtubule binding region of NDC80 [290], suggesting possible mislocalization that was, however, not experimentally addressed; within the tumor samples, FGFR2-NDC80 localized predominantly to the cell membrane [216].

NDC80 localizes to the centrosomes and mitotic spindles where it is necessary for assembly and stabilization of the kinetochore microtubules (reviewed in [290]). High NDC80 levels were found in cancers [291,292,293,294], and overexpression of NDC80 in mice led to abnormal spindle formation, hyperactivation of the mitotic checkpoint and initiation of the tumorigenic events [295]. Depletion or inhibition of NDC80 induced mitotic arrest, and suppressed xenograft tumor growth [294,296,297,298]. Taken together, these data suggest a possible involvement of mitotic defects in the FGFR2-NDC80 cancerogenesis, through ectopic FGFR and NDC80 activity.

### 4.5. FGFR2-CIT

Fusions of FGFR2 with the citron Rho-interacting kinase (CIT) were identified in non-small cell lung cancer and cholangiocarcinoma [215,217,218] (Table 1). FGFR2-CIT dimerized in cells, likely using the coiled-coil domain of CIT [149] (Figure 1), and induced oncogene addiction in Ba/F3 cells that was efficiently targeted by FGFR kinase inhibitors [267,299].

CIT functions in spindle orientation and during late cytokinesis [300,301,302,303]. CIT overexpression has been associated with cancers of various origin [304,305,306,307,308,309,310], likely through its kinase function that is, however, lost during chromosomal rearrangement in the FGFR2-CIT fusion (Figure 1). Transgenic mice expressing CIT variant lacking the kinase domain show defects in neurogenesis and spermatogenesis [311,312], due to aberrant cytokinesis that is followed by massive apoptosis. CIT also associates with primary cilia [313], and CIT downregulation inhibited ciliogenesis [314] and altered cilia length [315]. Therefore, it is possible that the FGFR2-CIT fusion produces CIT haploinsufficiency that may trigger cancerogenesis through cilia loss and mitotic defects.

### 4.6. FGFR2-OFD1

Fusions involving FGFR2 and the oral-facial-digital type 1 (OFD1) gene were reported in thyroid and endometrial cancer [149,219] (Table 1). FGFR2-OFD1 induced transformation of RK3E cells, that was abolished by FGFR kinase inhibitors [316]. Dimerization of the fusion protein likely occurs through the coiled-coil domains of OFD1 [149], which are preserved in the fusion protein (Figure 1), leading to transactivation of the FGFR2 kinase domain and activated ERK MAP kinase signaling [316].

OFD1 localizes to centrosome [317] where it is required for centriole maturation and primary ciliogenesis [318,319]. This localization requires the N-terminal part of OFD1 [320] that is, however, lost in the FGFR2-OFD1 fusion. Heterozygous loss-of-function mutations in OFD1 produce the OFD1 syndrome, an X-linked dominant disorder lethal in males that is characterized by systemic ciliopathy features [306,321,322,323,324]. The *Ofd1^+/−^* female mice reproduced the main patient phenotypes [318,325], suggesting haploinsufficiency in the heterozygous animals. The cilia were severely disrupted or lost, producing defects in laterality and Hh-dependent tissue patterning [318,326]. The zebrafish *ofd1* morphants also displayed laterality defects, due to cilia abnormalities in the Kupffer´s vesicle, as well as additional ciliopathy features [327]. These data suggest that the decreased levels of endogenous and centrosome-competent OFD1 in the FGFR2-OFD1 cancers may lead to deregulated ciliogenesis and cilia signaling, potentially contributing to neoplastic transformation.

### 4.7. FOP-FGFR1 

The type I fusion involving FGFR1 and the FGFR1 oncogene partner (FOP) is associated with a stem cell myeloproliferative disorder, acute myeloid leukemia (AML) [220,221,222,223,224,225,226,227] (Table 1). FOP-FGFR1 induced oncogene addiction in Ba/F3 cells [328,329,330], and transplanted FOP-FGFR1+ hematopoietic stem cells developed a fatal myeloproliferative disorder in mice [331].

FOP-FGFR1 comprises the leucine rich N-terminal region of FOP that facilitates dimerization and transactivation of the catalytic domain of FGFR1, and produces a constitutively active fusion protein [220,330,332] (Figure 1). Correspondingly, ERK MAP kinase and STAT signaling is increased in FOP-FGFR1 expressing cells [329,330]. Phosphoinositide 3-kinase (PI3K)/AKT pathway is also employed to sequester FOP-FGFR1 to the centrosome [328,330]. The mislocalization of FOP-FGFR1 [328,333,334,335,336] is also mediated by interaction with the centrosomal protein CAP350, through FOP [328,337]. The ectopic centrosomal FOP-FGFR1 activity then drives abundant cell division that was abolished by FGFR, PI3K and ERK pathway inhibitors [328,329,330,338]. The centrosomal localization appears critical for PLCγ phosphorylation [328,330,339,340] that is necessary for activation of the anti-apoptotic signaling in FOP-FGFR1 expressing cells [139,328,330,341]. Disruption of the PLCγ binding site delayed onset and prolonged survival of the mice transplanted with FOP-FGFR1 hematopoietic stem cells [331].

The FOP haploinsufficiency may contribute to FOP-FGFR1 cancerogenesis, as reduced FOP levels were shown to disrupt the centrosome structure and inhibit ciliogenesis [341,342,343], and similar defects were observed in FOP-FGFR1 expressing cells [227,340]. Although the hematopoietic cells do not produce cilia [344,345], the centrosome defects have also been associated with other myeloproliferative neoplasms [340,346], suggesting a common pathogenesis.

### 4.8. CEP110-FGFR1

The fusion of FGFR1 with the centrosomal protein 110 (CEP110) drives expansion of the hematopoietic stem cell population, and causes malignancies that frequently turn into AML [221,228,229,230,231,232,233,234,235,236,237,238,239,240,241,242,243,244,245,246,247,248] (Table 1). When expressed in cells, CEP110-FGFR1 likely dimerizes through the leucine zippers in CEP110 (Figure 1) which drives constitutive autophosphorylation of the FGFR1 kinase domains [247]. CEP110-FGFR1 induced oncogene addiction in Ba/F3 cells [241,347,348], that could be targeted by tyrosine kinase inhibitors [241,348]. Transplantation of murine bone marrow or human CD34+ cord blood cells transduced with CEP110-FGFR1 produced AML in the recipient mice [347], further supporting the role of CEP110-FGFR1 as an oncogenic driver.

Pluripotent stem cells derived from the AML^CEP110-FGFR1^ patient showed aberrant hematopoietic differentiation, which was restored by tyrosine kinase inhibitors; a growth inhibition was also achieved with isolated primary AML^CEP110-FGFR1^ cells [240]. This is in a sharp contrast with the clinical observation, as patients with CEP110-FGFR1 disease do not respond to tyrosine kinase inhibitors and have particularly poor prognosis; allogeneic hematopoietic stem cell transplantation appears the only viable option [238,349]. These data suggest that inhibition of the ectopic FGFR1 kinase activity in CEP110-FGFR1 cancers [241,350] does not bring clinical benefits, and that perhaps additional mechanisms contribute to the disease pathogenesis.

CEP110 is a structural protein of the centrosome [351,352], for which it requires a 170-aa region in the C-terminus that is retained in the CEP110-FGFR1 fusion (Figure 1) [247]. The centrosome localization of the fusion may, therefore, interfere with centrosome maturation, likely due to combination of the steric effects of the fusion and its ectopic kinase activity, which in turn produces centrosomal and spindle abnormalities and drives the oncogenesis [351,353,354].

## 5. Conclusions and Perspectives

The FGFR fusion proteins are oncogenic drivers; therefore, patients typically show a good initial response to the targeted therapy using FGFR tyrosine kinase inhibitors [171,186,215,219,269,270,355]. However, secondary gatekeeper mutations occur during therapy [270,356], and inhibition of effectors downstream from the FGFR oncogene has not delivered strong clinical benefit; therefore, alternate approaches are being developed. One such strategy takes advantage of the general overexpression of type II FGFR fusion proteins [268], which makes them a good target for cytotoxic conjugates specifically binding FGFR. For example, FGF2 conjugated with auristatin induced endocytosis of the FGFR1-FGF2/auristatin complexes, which released auristatin and produced a strong cytotoxic effect on cancer cells overexpressing FGFR1 [357]. Similarly, the FGFR-specific antibodies or antibody fragments conjugated to a cytotoxic molecule enter the cells via endocytosis to induce cell death [358,359]. Clinical trials evaluating cytotoxic conjugates in FGFR fusion-driven cancers are yet to emerge.

Another possibility is to specifically target the fusion protein. For example, no therapy protocol is available for FOP-FGFR1-driven cancers, which are very aggressive [221,222,328,331]. FOP-FGFR1 saturates at the centrosome, which appears critical for oncogenic transformation [329,331]. An adeno-associated virus-mediated delivery of interfering RNA, peptide or a coding sequence, specifically targeting the FOP-FGFR1 fusion or its interaction interface with the centrosome, therefore represents an attractive therapeutic possibility [360,361,362].

Finally, the ectopic activity of the FGFR fusion protein, together with decreased levels of the endogenous fusion partner, may contribute to neoplastic transformation through loss of primary cilia and deregulated cell division. Restoration of ciliogenesis and/or cilia function is, therefore, an attractive and so far unappreciated strategy to attenuate tumor growth. NSC12, an orally available analog of the naturally occurring FGF ligand trap pentraxin 3 (PTX3), was developed to target the FGF-driven pathologies [363]. NSC12 rescued ciliogenesis defects in three FGFR-driven cancer cell lines and a xenograft, and inhibited tumor growth [363]. The clinical studies evaluating cilia targeting as a cancer therapy are however yet to emerge.

## Figures and Tables

**Table 1:** FGFR fusion proteins in cancer.

Gene Fusion	Cancer Type (Cases)	Reference
FGFR3-TACC3	glioblastoma (29); NSCLC (28); HNSCC (11); bladder cancer (10); urothelial carcinoma (4); nasopharyngeal carcinoma (4); LUSC (4); glioma (3); ESCC (2); cervical cancer (2); gallbladder cancer (2); oral cancer (1); renal cell cancer (1); endometrial adenocarcinoma (1)	[133,149,153,179,185,186,187,188,189,190,191,192,193,194,195,196,197,198,199,200]
FGFR1-TACC1	low grade glioma (7); extraventricular neurocytoma (3); glioblastoma (2); spinal cord pilocytic astrocytoma (2); GIST (1)	[186,187,204,205,206,207,208,209,210,211]
FGFR2-BICC1	cholangiocarcinoma (47); hepatocellular cancer (1); colorectal cancer (1)	[149,175,177,212,213,214,215]
FGFR2-NDC80	cholangiocarcinoma (1)	[216]
FGFR2-CIT	NSCLC (3); cholangiocarcinoma (1)	[215,217,218]
FGFR2-OFD1	thyroid cancer (1); endometrial cancer (1)	[149,219]
FOP-FGFR1	AML (9); EMS (1)	[220,221,222,223,224,225,226,227]
CEP110-FGFR1	EMS (9); MPD (5); aCML (3); AML (2); CMML (1); AMML (1)	[221,228,229,230,231,232,233,234,235,236,237,238,239,240,241,242,243,244,245,246,247,248]

AML—acute myeloid leukemia; AMML—acute myelomonocytic leukemia; aCML—atypical chronic myeloid leukemia; CMML—chronic myelomonocytic leukemia; EMS—8p11 myeloproliferative syndrome; ESCC—esophageal squamous-cell carcinoma; GIST—gastrointestinal stromal tumor; HNSCC—head and neck squamous cell carcinoma; LUSC—lung squamous cell carcinoma; MPD—myeloproliferative disorder; NSCLC—non-small cell lung cancer.
